# Impact of Out-of-Pocket Cost on Herpes Zoster Vaccine Uptake: An Observational Study in a Medicare Managed Care Population

**DOI:** 10.3390/vaccines6040078

**Published:** 2018-11-21

**Authors:** Zhuliang Tao, Yong Li, Stephen Stemkowski, Kelly D. Johnson, Camilo J. Acosta, Dongmu Zhang, A. Mark Fendrick

**Affiliations:** 1 Comprehensive Health Insights, Inc., a Humana Co., 515 West Market Street Louisville, KY 40202, USA; zhuliangtao@gmail.com (Z.T.); yli4@humana.com (Y.L.); 2 Merck and Co., Inc. Center for Observational & Real World Evidence, 2000 Galloping Hill Rd, Kenilworth, NJ 07033, USA; kelly.johnson@merck.com (K.D.J.); zhangdongmu@hotmail.com (D.Z.); 3 Merck Research Laboratories, 2000 Galloping Hill Rd, Kenilworth, NJ 07033, USA; camilo_acosta2003@yahoo.com; 4 Center for Value-Based Insurance Design, North Campus Research Complex, 2800 Plymouth Road, Building 16/Floor 4/Room 455S, Ann Arbor, MI 48109-2800, USA; amfen@umich.edu

**Keywords:** out-of-pocket costs, herpes zoster vaccine, vaccination rate, utilization, abandonment

## Abstract

Herpes zoster (HZ) vaccination is approved for adults aged 50+ for the prevention of HZ, but it is underutilized. The objective of this study was to evaluate the association between out-of-pocket cost and HZ vaccine utilization. Adults aged 65 or older enrolled for at least 12 months in Medicare Advantage/Part D (MAPD) and Medicare Part D only (PDP) plans from 1 January 2007 to 30 June 2014 were selected. Abandonment was defined as a reversed claim for HZ vaccine with no other paid claim within 90 days. Out-of-pocket costs used were actual amounts recorded in the claim. Overall, the HZ vaccine abandonment rate was 7.3%. Mean out-of-pocket costs were higher for individuals who abandoned versus those who did not ($88 (±$55) versus $80 (± $49)). Logistic regression indicated individuals with out-of-pocket costs of $80–$90 were 21% more likely (OR = 1.21, 1.16–1.27 95% CI), and those with out-of-pocket costs >$90 were 90% more likely (OR = 1.90, 1.85–1.96 95% CI) to abandon than those with out-of-pocket costs <$80. The models also suggested that socioeconomic, racial, and ethnic disparities in vaccine abandonment existed. Different vaccine targeting efforts and pharmacy benefit design strategies may be needed to increase use, improve adherence, and minimize disparities.

## 1. Introduction

Herpes zoster (HZ), which is also known as shingles or zoster, is a manifestation of the reactivation of the varicella-zoster virus (VZV), which induces chickenpox as a primary infection. Following the initial VZV infection, the virus remains latent in sensory ganglia. In some individuals, latent VZV reactivates and replicates, perhaps as a consequence of waning cellular immunity, resulting in HZ [1,2,3,4,5,6,7,8]. Herpes zoster is a common disease with an overall annual incidence rate estimated at 4.5/1000 person-years in the United States (US) and ranging up to nearly 12.8/1000 person-years in adults aged 60 years and older [9]. As incidence increases with age, HZ and its complications are seen mostly in aged populations and may cause substantial acute and chronic morbidity [10,11]. The most common long-term complication of HZ is persistent pain, which is known as postherpetic neuralgia, which can persist for several months to years and may severely affect a patient’s quality of life [10]. Treatment options are available for patients with postherpetic neuralgia; however, the results of these treatments are limited. Prevention by immunization as recommended by the Centers for Disease Control and Prevention (CDC) might be viewed as potentially more effective and also more appealing to patients [12,13]. 

The single-dose, live HZ vaccine has been shown to be safe and effective, as demonstrated by Oxman et al. [14]. This large double-blind, placebo-controlled randomized trial with 38,546 immunocompetent persons 60 years of age and older showed that a single vaccination against HZ markedly reduced the incidence of HZ (5.42/1000 person-years in the vaccine group, versus 11.12 per 1000 person-years in the placebo group; an absolute difference of 5.70/1000 person-years). The vaccine also decreased the burden of illness due to HZ by 61% and the incidence of postherpetic neuralgia by 67% [14]. These and other such results have led to the HZ vaccine being recommended by the Advisory Committee on Immunization Practices (ACIP), which is a panel of medical and public health experts that develops vaccination strategies for public health. The ACIP recommendation advises a single dose of the vaccine for immunocompetent adults aged 60 years or older, regardless of whether or not they report a prior episode of herpes zoster [15]. While the live herpes zoster vaccine is no longer considered first-line treatment, it is still a recommended intervention. Although the U.S. Food and Drug Administration (FDA) approved the vaccine for use among persons aged 50 years and older without contraindications, the ACIP recommends vaccinations begin at the age of 60. According to recent estimates, only 24% of all adults aged 60 years or older received the vaccine, which is a level that falls well short of the Healthy People 2020 HZ vaccination goal of 30% [16].

The reasons for low HZ vaccination rates are thought to include, but are not limited to, financial barriers, lack of awareness, copay structure, lack of routine stocking of the vaccine, and one’s unwillingness to comply with the doctor’s advice [17]. Hurley et al. found that among internists and family medicine physicians, the most frequently reported barriers to vaccination were financial. Strikingly, only 45% of physicians knew that the HZ vaccine is reimbursed through Medicare Part D; and, of those who were receiving reimbursements, roughly 12% stopped administering the vaccine because of cost and reimbursement issues [17]. 

Information is lacking on the relationship between cost-sharing and use of the vaccine by beneficiaries with Medicare coverage. Since low vaccine adoption undermines public health efforts aimed at reducing the burden of herpes zoster, we examined the factors associated with HZ vaccine use among individuals enrolled in Medicare Advantage/Part D (MAPD) and Part D only (PDP) plans.

## 2. Materials and Methods

### 2.1. Data Source 

Individuals aged 65 or older, as well as certain younger people with disabilities, are eligible for the Medicare program in the United States. Medicare Advantage (MA) plans are health plans that are approved by Medicare, and offered and administered by private insurers. These plans are required by law to cover all hospital and physician services that the original Medicare plan covers. Many MA plans also include prescription drug coverage, i.e., MAPD. Stand-alone prescription drug plans (PDP), are also available. Humana is the one of the largest providers of MAPD and PDP plans in the United States. This retrospective observational cohort study used data collected from Humana, Inc. (Louisville, KY, USA) health plan claims for the period 1 January 2007 through 30 June 2014. The data sources for this study included member enrollment, medical, and pharmacy claims data and health plan benefit design data that were linked to each member via a unique patient identifier, plan benefit package code, and formulary identifier. Socioeconomic and additional demographic data were obtained from AmeriLINK^®^ market data, licensed by Humana, and matched to the research data at the member level by a third party using a unique, randomly generated member identifier under the appropriate protective agreements necessary to confirm a positive link. The study protocol and relevant supporting information were approved by Schulman and Associates, which is an independent Institutional Review Board (Protocol No. 0000343). The process for de-identifying datasets complied with the de-identification standard in the HIPAA privacy rule, as outlined in CFR 164.514. 

### 2.2. Selection of Subjects

Individuals enrolled in a Medicare Advantage/Part D (MAPD) or Medicare Part D only (PDP) health plan, and aged 65 or older who were eligible for the herpes zoster vaccine based on indicated age between 1 January 2007 and 30 June 2014 were identified for study inclusion. Though the vaccine is indicated for adults aged 60 years or older, vaccine coverage for adults aged 60–64 is addressed differently from Part D requirements regarding the elements of vaccine administration and cost-sharing considerations. 

The study period encompassed 1 January 2007 through 30 June 2014. The identification period was 1 April 2007 through 30 June 2013. Individuals who had any pharmacy claims for the zoster vaccine were selected, and the first observed date for the vaccine recorded was designated as the index date. A 90-day pre-index period and a 365-day post-index period were established for the analysis. Members with pharmacy coverage but no prescription drug utilization history, members with dual eligibility for Medicare and Medicaid, low-income subsidy recipients, and members with evidence of receiving the HZ vaccine prior to age 65 were excluded from the study. Members aged 90 or older at index were excluded from all of the analyses.

### 2.3. Primary Study Measures

Vaccine use was identified by National Drug Code and Generic Product Identifier (GPI) codes. Vaccine abandonment was a dichotomous variable indicating whether or not the individual fulfilled the vaccine prescribed by their physician. An individual was considered to have abandoned the vaccine when a claim record indicated that a HZ vaccine order was never fulfilled or had been reversed, and no other paid record of the vaccine was observed in the 90-day period following the non-fulfillment of the index vaccination. Vaccine abandonment rates were calculated as the ratio of the number of people who abandoned their HZ vaccine to the number of people who had a claim for the vaccine. Rates were calculated using both electronically-transmitted and traditionally processed pharmacy claims, i.e., paper claims. 

Member out-of-pocket amounts were the actual member OOP cost in the claim record, in US dollars ($) from the prescription claim in which the HZ vaccine was reported. This value represented the actual amount for which the member was responsible based on the accrued applicable deductibles and copayment requirements in place at the time the claim was incurred. We capped the maximum OOP amount at the average wholesale price plus 20% ($275) to address obvious outlier values (less than 1% of cases). All of the members were classified into these mutually-exclusive categories of OOP expense amounts reported in the claim: $0–$80, >$80–<$90, and $90 or more to reflect distribution characteristics of the OOP amounts.

### 2.4. Other Relevant Measures

Other measures of interest included demographic and clinical characteristics during the pre-index period. Age at index, gender, and geographic region were determined from enrollment data. Race was available in the Center for Medicare and Medicaid Service’s (CMS) enrollment data for patients with Medicare coverage. Region was based on the state of residence, grouped into regions according to census classifications. To determine community type, we divided the population into three categories: rural, urban, and suburban. Population density was assigned to a community type class by matching member zip codes to Rural–Urban Commuting Area (RUCA) codes and applying the Washington State Department of Health’s RUCA consolidation system as follows: (1) Urban (50,000 persons or more); (2) Suburban (small cities/large towns); (3) Rural (small towns with populations below 10,000 or isolated rural areas).

Estimated household income was derived from financial survey data at the person level by AmeriLINK^®^. Education levels were also provided by AmeriLINK^®^ based on self-reported responses and 2010 census data. Comorbidity burden was measured by the RxRisk-V score, which is a prescription claims-based comorbidity index originally developed for use in the Veterans Health Administration (VHA) population [18]. The score was determined based on the identification of 45 distinct comorbid conditions via their associated medication treatments. To calculate the RxRisk-V score, comorbid conditions were mapped to drug classes and individual drugs via Medi-Span GPI codes. By construction, the RxRisk-V score can range from 0 to 45, with a higher score indicating a greater number of comorbid conditions.

### 2.5. Statistical Analysis

The study examined HZ vaccine adoption by assessing the extent of vaccine abandonment among those who received prescriptions for it. Descriptive analyses of vaccine use were conducted to estimate the overall abandonment rate. Results were stratified and reported by member OOP cost categories. The multivariable abandonment analyses used odds ratio estimates and 95% confidence intervals from logistic regression models to identify key factors related to vaccine non-compliance. The outcome variable of the logistic models was the likelihood of abandoning a HZ vaccine order. Factors related to household income, educational attainment, coverage type, gender, age, regional differences, and patient share of the cost of the vaccine were described and introduced into multivariable models to estimate the impact of these factors. Community type (rural, suburban, or urban), Medicare plan type, and RxRisk-V score were included in the analysis as covariates. Covariates were evaluated for collinearity before entering into the models. Model fit was assessed via C statistic.

## 3. Results

Overall, 6,295,970 individuals met the selection criteria and were included in the analysis. Of these, 2,547,652 (40.5%) were enrolled in MAPD, and 3748.318 (59.5%) were enrolled in PDP. Females comprised 59.0% of the sample overall, which was a pattern that was reflected in the MAPD and PDP cohorts individually (Table 1). Mean age was reported at 72.6 (±6.1, standard deviation) years and 73.4 (±6.6) years in the MAPD and PDP cohorts, respectively. Individuals from the Midwest and south comprised 85% of the MAPD cohort, but only 65% of the PDP sample. Nearly two-thirds of the sample were in households with less than $45,000 annual household income. Half reported at least some college education. The mean RxRisk-V comorbidity index was similar for MAPD (5.10 ± 2.86) and PDP (5.02 ± 2.95) members.

With regard to vaccine abandonment rates, we found that for those individuals with a claim for the HZ vaccine, (*n* = 192,606, 35% MAPD, *n* = 357,623, 65% PDP, Table 2), 8.9% of MAPD and 6.4% of PDP members did not fulfill the vaccine. This represents an overall abandonment rate for the vaccine of 7.3% for Medicare members. Interestingly, members in PDP plans, despite higher OOP amounts, reported fewer abandoned HZ vaccine prescriptions than did members in MAPD. The mean OOP cost for members in MAPD plans who abandoned the vaccine was $79.76 (± $45.51) compared with $71.35 (± $40.95) for members who did not. Similarly, mean OOP amounts for PDP members who did not fulfill the vaccine order was $94.52 (± $61.08) versus $84.83 (± $52.17) for members who did. 

Three multivariable analyses were conducted (MAPD, PDP, and combined) to understand the key factors related to the likelihood of a patient to abandon the HZ vaccine. Overall, and by Medicare plan type, female members had a 4–7% lower odds of abandoning the HZ vaccine than male members. Increasing age also was significantly associated with abandonment. Black and Hispanic members were found by all three models to be more likely than white members to abandon the vaccine. 

Relative to those in the northeast, individuals in the south had 40% higher odds, and individuals in the Midwest 11% lower odds, of abandoning the HZ vaccine. Compared to suburban members, urban members were more likely, while rural members were less likely, to abandon (Table 3).

We observed an inverse relationship between the likelihood of vaccine abandonment and educational attainment level. Individuals with a high school diploma had 16% higher odds, and those with less than a HS diploma had 33% higher odds of abandoning the vaccine than individuals with at least some college education. Similarly, individuals with household incomes between $45,000 and $87,000 had 23% higher odds of abandoning than those whose income was greater than $87,000. Individuals whose incomes were less than $45,000 had nearly 50% higher odds of abandonment. Individuals with a higher comorbidity index also were more likely to abandon (Table 3).

Compared to those with an OOP amount less than $80, the odds of abandonment was 21% higher among individuals whose OOP amount was between $80–90, and 90% higher among individuals whose OOP amount was greater than $90. Putting this in perspective, among individuals with an OOP amount less than $80, about 6.9% did not fulfill their HZ vaccine order, giving estimated odds of 0.074. Based on the logit models, an OOP amount between $80–90 would increase the odds of abandonment to 0.090, and an OOP amount greater than $90 would increase the odds of abandonment to 0.141. Despite the relatively higher OOP amounts, members enrolled in PDP had 16% lower odds of abandoning the HZ vaccine than did members in MAPD plans. (Table 3).

## 4. Discussion

These study results suggest the importance of certain socioeconomic and demographic factors linked to the likelihood that individuals will be vaccinated when the HZ vaccine is prescribed, recommended, or sought out. These results also mirror those found in studies of HZ vaccination, and other vaccines in which non-white, less affluent, and less highly educated people reported lower vaccination rates than other socioeconomic and demographic groups [19]. Hechter, et al. (2013) [20] reported similar patterns of disparity in a study of eligible health plan members and utilization of the HZ vaccine. Although they did not specifically examine the impact of member OOP amount related to the vaccine abandonment, they suggested the importance of cost to the patient as a factor related to vaccine utilization [20]. 

Furthermore, other studies have shown that vaccine abandonment is associated with: (a) financial barriers (e.g., cost-sharing and inadequate reimbursement) [14,21,22]; (b) financial barriers to pharmacies to stock a wide range of recommended vaccines [14]; and (c) insurance benefit design related to coverage of immunizations [23,24]. Interventions that reduce patient cost-sharing have reported a broad range of increases in immunizations (1–47%), with the enhanced vaccination rates dependent on the magnitude of the cost-sharing reduction [25,26].

Similarly, the present study results also support the notion that member OOP cost is significantly associated with vaccine compliance. As the amount of OOP cost increased, vaccine abandonment was more likely. For most people in the analysis, the copayment for the vaccine fell between $80–90. Utilization rates above and below these levels exhibited the expected pattern; the lower OOP group had a lower probability of abandoning the vaccine, the higher OOP group reported a higher probability of abandonment. In studies of non-adherence focusing on specialty medications, OOP cost was a significant factor in poor adherence and primary abandonment, which may be a factor in this study [27]. 

Comparing Medicare coverage types also produced interesting differences with regard to cost. Most notable was the difference between MAPD and PDP members. There was 16% lower odds of abandonment for PDP members than MAPD members. 

The limitations in this study included the use of pharmacy data that contained both electronically transmitted and traditionally ordered vaccines. The traditional transmission of orders did not account for members who received the order but never pursued the vaccine. As a result, abandonment rates for traditionally-transmitted orders may be underestimated. Though not as likely to be offered to patients in as many and varied places as other vaccines such as flu, the possibility exists that a patient may have been vaccinated outside of their primary insurance program or may have been vaccinated prior to enrolling, or after leaving the health plan, which may have affected the estimates. Despite efforts to identify HZ vaccine use prior to the index date, it is possible that individuals with no recorded claim for the vaccine in the study data may have received it under prior coverage. 

The limitations that are common to studies using administrative claims data applied to this study also. These included a lack of certain information in the database and errors in claims coding. No causal inference may be ascertained from this study, as it was an observational study using retrospective claims data. Since this study used data from Humana members only, the results may not be generalized to the general population; however, Humana is a large national health plan with members residing in a broad array of geographic regions.

## 5. Conclusions

The results related to socioeconomic and demographic factors indicated that disparities based on income, education, and race/ethnicity regarding vaccine access and abandonment exist. Patient OOP cost was also an important factor influencing HZ vaccine adoption in individuals enrolled in Medicare. Increased OOP amounts are associated with an increased risk of abandonment. Cost-sharing continues to be an issue for vaccines covered under Medicare Part D, including the HZ vaccine. Programs that improve access to and distribution of the vaccine to a broader segment of the population at risk may improve vaccination rates, minimize disparities, and enhance patient-centered outcomes. Efforts to reduce OOP cost of the HZ vaccine, such as formulary calibration, copay adjustment or patient subsidies, may also help improve use and adherence of HZ vaccine.

## Figures and Tables

**Table 1:** Baseline Demographic Characteristics of Herpes zoster (HZ) Vaccine Eligible Adults by Coverage Type. MAPD: Medicare Advantage/Part D.

Eligible Members (Count)	Medicare MAPD		Medicare Pt D		All	
	2,547,652		3,748,318		6,295,970	
	*N*, or Means	%, or SD	*N*, or Means	%, or SD	*N*, or Means	%, or SD
Gender (count, %)						
Female	1,432,202	56.0	2,281,911	61.0	3,714,113	59.0%
Male	1,115,450	44.0	1,466,407	39.0	2,581,857	41.0%
Age (Mean, SD)	72.56	6.14	73.42	6.58	73	6.00
Geographic Region (count, %)						
Midwest	601,217	24.0	1,008,646	27.0	1,609,863	25.6%
Northeast	67,250	3.0	523,248	14.0	590,498	9.4%
South	1,565,019	61.0	1,417,258	38.0	2,982,277	47.4%
West	296,017	12.0	777,838	21.0	1,073,855	17.1%
Unknown	18,149	1.0	21,328	1.0	39,477	0.6%
Race/Ethnicity (count, %)						
White	622,729	24.0	781,387	21.0	1,404,116	22.3%
Black	97,593	4.0	60,987	2.0	158,580	2.5%
Hispanic	13,444	1.0	27,060	1.0	40,504	0.6%
Other	27,766	1.0	64,687	2.0	92,453	1.5%
Unknown	1,786,120	70.0	2,814,197	75.0	4,600,317	73.1%
Population Density (count, %)						
Rural	296,760	12.0	659,061	18.0	955,821	15.2%
Suburb	624,428	25.0	960,385	26.0	1,584,813	25.2%
Urban	1,608,315	63.0	2,107,544	56.0	3,715,859	59.0%
Unknown	18,149	1.0	21,328	1.0	39,477	0.6%
Household Income ($)						
≤$45K	1,656,774	65.0%	2,414,518	64.4%	4,071,292	64.7%
45 K and $87.5 K	434,298	17.0%	560,368	14.9%	994,666	15.8%
$87.5 and $112 K	219,535	8.6%	320,330	8.5%	539,865	8.6%
≥$112K	237,045	9.3%	453,102	12.1%	690,147	11.0%
Attained Education	8,531	0.3%	17,652	0.5%	26,183	0.4%
HS diploma	976,142	38.3%	1,112,675	29.7%	2,088,817	33.2%
Any college	1,283,238	50.4%	2,015,512	53.8%	3,298,750	52.4%
Unknown	279,741	11.0%	602,479	16.1%	882,220	14.0%
RxRiskV (mean, SD)	5.10	2.86	5.02	2.95	5.05	2.92

**Table 2:** Utilization of HZ Vaccine in Eligible Adults by Coverage Type. OOP: out of pocket.

Eligible Members 2007–2014 (Count)	Medicare MAPD		Medicare Pt D		All	
	2,547,652		3,748,318		6,295,970	
	*N*, or Means	%, or SD	*N*, or Means	%, or SD	*N*, or Means	%, or SD
Overall abandonment (N, rate %)						
Fulfilled	175,506		334,628		510,561	
Abandoned	17,100	8.9	22,995	6.4	40,139	7.3
eRx abandoned (N, rate %)						
Fulfilled	20,242		32,763		53,005	
Abandoned	8,160	28.7	9,312	22.1	17,472	24.8
Trad. abandoned (N, rate %)						
Fulfilled	153,308		296,492		449,800	
Abandoned	8,742	5.4	13,103	4.2	21,845	4.6
OOP among fulfilled ($, mean, SD)	71.35	40.95	84.83	52.17	80.21	49.03
OOP among abandoned ($, mean, SD)	79.76	45.51	94.52	61.08	88.24	55.45
Total OOP ($, mean in 12 months, SD)	419.9	661.87	577.94	866.09	515.25	795.15

**Table 3:** Logistic Regression on Abandonment of HZ Vaccine among MAPD and prescription drug plans (PDP) Members.

Effect	Odds Ratio	95% Confidence Limits		*P* Value
Female (ref: male)	0.96	0.94	0.99	0.001
Age	1.01	1.01	1.01	<0.001
Race (ref: White)				
Black	1.58	1.47	1.69	<0.001
Hispanic	1.48	1.40	1.57	<0.001
Other race	0.97	0.95	1.00	0.053
OOP cost category (ref: $0–80)				
OOP cost category (>$80 and <$90)	1.21	1.16	1.27	<0.001
OOP cost category (≥$90)	1.90	1.85	1.96	<0.001
Service Year (ref: 2007)				
2008	1.18	1.11	1.27	<0.001
2009	1.60	1.50	1.70	<0.001
2010	1.51	1.40	1.62	<0.001
2011	1.17	1.10	1.25	<0.001
2012	1.41	1.33	1.50	<0.001
2013	1.37	1.29	1.45	<0.001
2014	2.20	2.07	2.34	<0.001
PDP (ref: MAPD)	0.84	0.82	0.86	<0.001
Geographic area (ref: Northeast)				
Midwest	0.89	0.85	0.93	<0.001
South	1.40	1.34	1.47	<0.001
West	0.96	0.91	1.01	0.103
Community type (ref: Suburban)				
Urban	1.20	1.17	1.23	<0.001
Rural	0.92	0.88	0.95	<0.001
Education (ref: Any college)				
Below high school	1.33	1.29	1.37	<0.001
High school	1.16	1.12	1.20	<0.001
Household income (ref: High income)				
Low income (<$45,000)	1.48	1.25	1.75	<0.001
Mid income ($45,000–87,000)	1.23	1.20	1.26	<0.001
Disease Burden				
RxRiskV index	1.07	1.06	1.07	<0.001

Notes: Outcome: abandoned = 1 vs. fulfilled = 0 (vaccinated). Observations: 499,491 (36,550 abandoned, 462,941 fulfilled).
